# Calcium Nanoparticles Impregnated With Benzenedicarboxylic Acid: A New Approach to Alleviate Combined Stress of DDT and Cadmium in *Brassica alboglabra* by Modulating Bioacummulation, Antioxidative Machinery and Osmoregulators

**DOI:** 10.3389/fpls.2022.825829

**Published:** 2022-03-09

**Authors:** Samavia Mubeen, Iqra Shahzadi, Waheed Akram, Wajid Saeed, Nasim Ahmad Yasin, Aqeel Ahmad, Anis Ali Shah, Manzer H. Siddiqui, Saud Alamri

**Affiliations:** ^1^State Key Laboratory for Biocontrol, School of Life Sciences, Sun Yat-sen University, Guangzhou, China; ^2^Hubei Key Laboratory of Biomass Resource Chemistry and Environmental Biotechnology, Hubei International Scientific and Technological Cooperation Base of Sustainable Resource and Energy, Hubei Engineering Center of Natural Polymersbased Medical Materials, School of Resource and Environmental Science, Wuhan University, Wuhan, China; ^3^Department of Plant Pathology, Institute of Agricultural Sciences, University of the Punjab, Lahore, Pakistan; ^4^Key Laboratory of Crop Cultivation and Farming System, Agriculture College, Guangxi University, Nanning, China; ^5^Senior Superintendent Garden, University of the Punjab, Lahore, Pakistan; ^6^Guangdong Key Laboratory for New Technology Research of Vegetables/Vegetable Research Institute, Guangdong Academy of Agricultural Sciences, Guangzhou, China; ^7^Key Laboratory of Land Surface Pattern and Simulation, Institute of Geographic Sciences and Natural Resources Research, Chinese Academy of Sciences, Beijing, China; ^8^Department of Botany, Division of Science and Technology, University of Education, Lahore, Pakistan; ^9^Department of Botany and Microbiology, College of Science, King Saud University, Riyadh, Saudi Arabia

**Keywords:** abiotic stress, bioaccumulation, *Brassica alboglabra*, cadmium uptake, DDT, heavy metal removal, organochlorine, persistent organic pollutant

## Abstract

At present, the alleviation of stress caused by climate change and environmental contaminants is a crucial issue. Dichlorodiphenyltrichloroethane (DDT) is a persistent organic pollutant (POP) and an organochlorine, which causes significant health problems in humans. The stress caused by cadmium (Cd) and the toxicity of DDT have direct effects on the growth and yield of crop plants. Ultimately, the greater uptake and accumulation of DDT by edible plants affects human health by contaminating the food chain. The possible solution to this challenging situation is to limit the passive absorption of POPs into the plants. Calcium (Ca) is an essential life component mandatory for plant growth and survival. This study used impregnated Ca (Bd_Ca_) of benzenedicarboxylic acid (Bd) to relieve abiotic stress in plants of *Brassica alboglabra*. Bd_Ca_ mitigated the deleterious effects of Cd and reduced DDT bioaccumulation. By increasing the removal efficacy (RE) up to 256.14%, Bd_Ca_ greatly decreased pollutant uptake (Cd 82.37% and DDT 93.64%) and supported photosynthetic machinery (86.22%) and antioxidant enzyme defenses (264.73%), in applied plants. Exogenously applied Bd also successfully improved the antioxidant system and the physiochemical parameters of plants. However, impregnation with Ca further enhanced plant tolerance to stress. This novel study revealed that the combined application of Ca and Bd could effectively relieve individual and combined Cd stress and DDT toxicity in *B. alboglabra*.

## Introduction

Dichlorodiphenyltrichloroethane (DDT), an organochlorine and a persistent organic pollutant (POP), was first synthesized in 1874 to control insect pests on large scales in the agriculture fields (Turusov et al., 2002). Its effectiveness evolved into the most abundantly used pesticide before it was banned by the Environmental Protection Agency (EPA) in the United States during 1972 (Jaga and Dharmani, 2003). It was famous for the long-term pest control, more effectiveness, easy manufacturing, and cost-effectiveness (Sadasivaiah et al., 2007). Besides, DDT is also released as a by-product of all the chemical industries dealing with or producing chlorinated chemicals/liquids. Because DDT has been used in bulk amounts worldwide and for long periods, it has become a high-ranked source of toxicity for the human population without any geographical discrimination. Some other chemical processes that involved chlorine (e.g., smelting of metals, bleaching of paper pulp, production of cement, impregnation of wood, and incineration of solid waste and sewage sludge) add a significant share in the DDT contents in the environment.

Cadmium (Cd) is among the most injurious metals imposing multiple detrimental effects on the growth and development of plants and concomitantly on humans (Sardar et al., 2022a). Rapid industrialization and excessive use of Cd-based fungicides, herbicides, insecticides, and fertilizers are continuously contaminating soils and water (Sardar et al., 2022b). Cd-contaminated soils disturb enzymatic activities, metabolic processes, plant growth, and quality of the plant produce (Shah et al., 2021). The consumption of Cd-contaminated plant commodities may cause gastrointestinal and pulmonary disorders besides cancer. Soil Cd uptake is a primary source of food chain contamination. Cd is bioaccumulation in the foliage (> 5–10 μg g^–1^) and has harmful effects on the physiochemical activities of plants. Henceforward, it becomes indispensable to adopt some suitable strategies to lower Cd toxicity and reduce its uptake by plants growing in polluted sites.

Both Cd and DDT are readily absorbed in plant tissues, translocated between different tissues, and preserved in the lipid-rich areas. Complete avoidance of these pollutants is necessarily important, which seems impossible if the pollutants are part of our everyday diet. Therefore, limiting Cd and DDT contents in agri-food products can greatly assist in avoiding pollutant bioaccumulation in human tissues.

*Brassica* is an oil seed-producing plant famous for its consumption as a leafy vegetable and animal feed. Vegetables are perishable crops, therefore, mostly cultivated in agricultural lands situated near urban areas. In such regions, crops may be irrigated by water contaminated with industrial wastewater containing Cd and other metal contaminants (Rezapour and Samadi, 2011). Cost-effective DDT has been extensively used for the management of insect pests of several crops. Residues of DDT are still present in these soils (Mansouri et al., 2017). Growing such an important crop in a DDT- and Cd-polluted environment causes the pollutants to be absorbed, translocated, and preserved in the edible plant parts. Plant pollutants cause orchestration in the concentration of total soluble protein, soluble sugars, leaf water, and osmoprotectants besides the decline in the activity of antioxidant enzymes, including ascorbate peroxidase (APX), superoxide dismutase (SOD), and peroxidase (POD) in stressed plants (Yasin et al., 2018a; Ahmad et al., 2020b; Li et al., 2021). They severely affect plant growth, photosynthesis, chlorophyll activity, antioxidant activity, reactive oxygen species (ROS), and many other physiological attributes.

Benzenedicarboxylic acid (Bd) is well-documented for driving plant physiological responses toward a new equilibrium. It successfully induced antioxidant defenses, improved enzymatic activities, and augmented the nutritional quality of fruits (Ahmad et al., 2019). Considering its diverse physiological effects in plants, and potential to improve fruit quality, Bd was selected to inhibit DDT and Cd absorption from the surrounding environment. This is the first study that evaluates the molecule in limiting the absorption and translocation of plant stressors. Nevertheless, Bd is readily soluble in water. Therefore, it can be easily scavenged or washed off from plant surfaces during rain. Its molecules contain chromophores (with absorbance at wavelengths >290 nm), therefore, stay highly susceptible to photolysis by sunlight, and also degrade readily in water (Lu et al., 2002). Consequently, its stable, slow, and continuous release needs some safeguard agent for which calcium (Ca) impregnation could be a useful strategy.

Calcium nanoparticles are a family of inorganic layered materials (Ram Reddy et al., 2006; Xu et al., 2006; Reddy et al., 2008), which can be synthesized using a cost-effective protocol (Xu et al., 2006). The main feature of Ca includes its role in the formation of plant structure. The Ca can be applied to plants for increased growth and defense parameters (Thor, 2019). Several positive physiological impacts have already been documented on Ca application (Fletcher et al., 2020). Ca alleviates Cd and other abiotic stresses in applied plants (Sakouhi et al., 2021; Tong et al., 2021).

To reduce the accumulation level of Cd and DDT in crops, to ensure food safety, and to reduce human intake, this study found an additive that can effectively reduce the absorption of pollutants by crops. It was hypothesized that the combined application of Ca with Bd might have beneficial effects on plant stress alleviation and growth improvement. According to our information, there is no study demonstrating the role of Bd and/or Ca in alleviating multiple environmental stresses. Henceforth, this study is designed to investigate the role of Bd-coated Ca (Bd_Ca_) in alleviating multiple stress (DDT and Cd) conditions in Kale plants and their joint effect of controlling the bioaccumulation of the toxic organic contaminant. This study elucidates the role of Bd_Ca_ on growth, gas exchange attributes, and antioxidative system of *Brassica alboglabra* plants under DDT and temperature stress. Moreover, this study was deliberated to examine the potential of Bd_Ca_ in reducing the bioaccumulation of toxic organic contaminant, i.e., DDT.

## Materials and Methods

### Plant Material and Growth Conditions

Seeds of *B. alboglarba* were surface sterilized by submerging in 0.1% potassium permanganate for 15 min and subsequent thorough washing by using deionized water. The 10 sterilized seeds were placed on 2 layers of 3-mm Whatman filter paper embedded in 9 cm glass jars supplemented with 10 ml of standard Davtyan (0.75 N) nutrient solution in ddH_2_O (Daryadar et al., 2019). All aforementioned steps were performed beneath very low light. Then, these covered jars were randomly kept in a plant growth chamber in the dark at 25 ± 2°C for 5 days. After the growth of the initial 5 days, seedlings were transferred to pots (2 plants/pot) packed with 1 kg of Cd-contaminated UQ23 pot soil.

### Bd_Ca_ Synthesis

The method by Mitter et al. (2017) was strictly followed to prepare Ca nanoparticles. The process included non-aqueous precipitation, heat treatment, purification step, and dispersion in water. The complete procedure yielded an average particle size of 45 nm, which were analyzed by a Nanosizer, Nano ZS instrument (Malvern Instruments) to obtain the *Z* average size and particle diameter (PdI). The chemical composition and crystal structure were verified by powder X-ray diffraction (XRD) with five Ca samples (Rigaku Miniflex X-Ray Diffractometer), Fourier-transform IR spectroscopy (Nicolet 6700 FTIR; Thermo Electron Corporation) with attenuated total reflection mode (Xu et al., 2006), and imaged by JEOL Transmission Electron Microscope, JSM-2010 (Supplementary Data Set 1).

### Loading of Bd

To define optimal and complete loading of respective Bd into Ca nanoparticles, the ratio of Bd/Ca was adjusted to 1:10. A mixture of 10 ml of Bd and 100 ml of Ca was prepared and incubated at room temperature for 30 min with continuous agitation of 120 rpm. The quantification of the residual Bd was carried out using the standard method developed by Sun et al. (2020). High-performance liquid chromatography (HPLC) system equipped with ZORBAX-Eclipse XDB-C18 column (4.6 mm × 250 mm, 5 μm, Agilent) separated the free Bd molecule aqueous mobile phase of 40% methanol flowing at the rate of 0.4 ml min^–1^. Samples of 10 μl were separately processed at 40°C, and absorbance readings were recorded at 210 nm. The stable release of Bd was also ensured at three different incubation temperatures of 0°C, 25°C, and 50°C for a period of 60 by using a method as described earlier (Supplementary Data Set 1).

### Treatment Application

Bd (C_19_H_22_O_6_) was purchased from the RPI Research Products International, CAS # 77-06-5. The growth regulator was initially dissolved in ethanol to prepare a stock concentration of 0.5 mM L^–1^ and then diluted with distilled sterilized water to attain a concentration of 5 μM L^–1^ as a working concentration recommended by Miao et al. (2017). Similarly, the Bd_Ca_ solution was prepared to get the final working concentration of 0.5 mM L^–1^ for Bd. However, the concentration of Ca was 1 g L^–1^. The third treatment of Ca nanoparticles was also prepared (1 g L^–1^). Each seedling received 0.5 ml of the respective spray treatment.

To elucidate the pathway-specific uptake of DDT into the plants, the experiment was designed to expose potted *B. alboglabra* plants to DDT through the use of incubation chambers (ICs). The ICs were designed and built using glass material by strictly following the method of a previous study (Zhu et al., 2016). There were two separate entry points for air and DDT into the chamber, powered with electric pumps and connected through polytetrafluoroethylene pipes. Chambers also have an air sampling system and a temperature regulation system. A separate chamber was used for each treatment. The potting soil was covered with a 2-cm layer of silica sand (150-380 μm) to minimize the DDT exchange between the soil and the air (Zhu et al., 2020). The DDT concentration in the air was adjusted to 1,000 mg m^–3^ as derived from its minimum toxic concentration of 582.4 mg m^–3^ and kept constant throughout the incubation period (Jarrell et al., 2002). To compensate for the loss of particulate on the plant surface over time, the POP dust-spraying step was repeated weekly. Later, the conditions of ICs were adjusted at 70-76% relative humidity and 500–550 mmol m^–2^ s^–1^ light with a 16-h photoperiod. Five replications were used for each treatment. Cd treatment was applied to the plants by strictly following the method developed by Li et al. (2021). All these dose treatments were adopted based on the previous literature and the highest quantity of pollutants recorded in the environment. Furthermore, the concentration of the pollutants was kept stable in the growth chamber.

### Determination of Growth, Physiological, and Antioxidant Defense Parameters

After 8 weeks of incubation, entire plants were sampled randomly. The harvested plants were separated into the root, shoot, and leaf samples. These plant parts were kept individually in distilled water for 60 s. Later, plant parts were dried on blotting paper and stored at −80°C. The plant samples were processed for the estimation of Leaf Relative Water Content (LRWC), Photosynthetic pigments (Chl *a* and Chl *b*), Soluble Sugars, Gas Exchange Parameters, net Photosynthetic Rate (*Pn*), Estimation of Cd Contents, Metal Tolerance Index (MTI), Evaluation of Hydrogen Peroxide, Lipid Peroxidation, Electrolyte Leakage (EL), Leaf Osmotic Potential, Membrane Stability Index (MSI), Estimation of Water Potential, Proline Content, β-carotenoids Contents, and the parameters of Oxidative and Antioxidant Enzymatic Activities using the standard optimized protocols (Khan et al., 2019).

### Quantification of DDT

The DDT was quantified by gas chromatography-mass spectrometry (GC-MS), as described by Baumer et al. (2020). The system consisted of GC-MS (QP) by Shimadzu (Kyoto, Japan), coupled with a mass detector and a capillary column. Detailed specifications were 30 m DB-5, 0.1 μm film thickness, and 0.25 mm internal diameter of the capillary column used for the determination of DDT. Samples were injected using a splitless injection mode while keeping an ion source temperature of 250°C and using helium as the carrier gas at 0.75 ml/min flow rate. The oven temperature was ramped at the rate of 4°C min^–1^, initializing its range from 120°C to 190°C, and at the rate of 32°C min^–1^ up to 270°C, which was kept constant for 4 min. By operating the mass spectrometer at scanning mode between 45 and 475 Da (*m*/*z*), DDT was detected in the sample size of 50 μl with a minimum limit of 1 mg kg^–1^.

### Calculation of Mobility, Distribution, and Remediation Efficiency

The translocation factor (TF) and avoidance efficiency (AE) were used to evaluate the ability of the plants to absorb, translocate, and/or avoid pollutants, according to the following equations (Xiao et al., 2019; Zeng et al., 2019):

(1) TF = C_substance shoot_/C_substance leaf_
(2) AE = (C_substance shoot_ × M_shoot_ × C_substance leaf_ × M_leaf_)/ (C_substance air_ × M_air_)

where C_substance shoot_, C_substance leaf_, and C_substance air_ are the contents of DDT (mg⋅kg^–1^) in shoots, roots, and air, respectively. However, the mass of one cubic meter of air was standardized as 1.29 kg during the experimentation.

### Statistical Analysis

Randomized one-way ANOVA was used for the comparisons between different treatments. Means were compared using the all pairs Tukey’s honestly significant difference test. Statistical calculations of ANOVA were performed using DSAASTAT statistics software.

## Results

### Evaluation of Bd_Ca_

**FIGURE 1:**
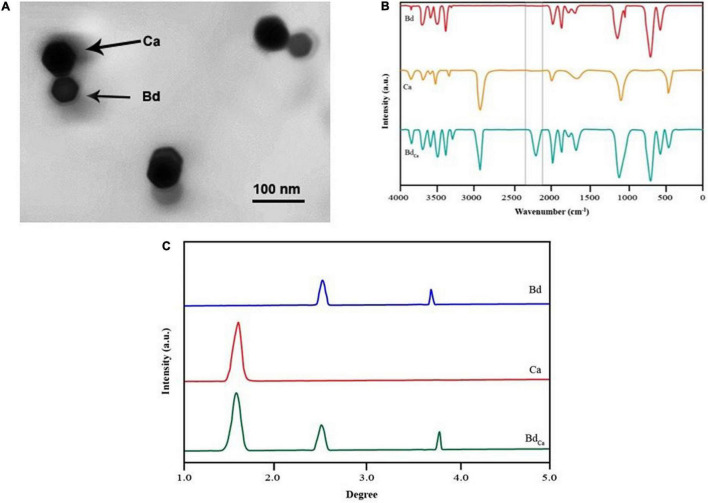
Transmission elctron micrograph of Calcium (Cal) particles coupled with benzenedicarboxylic acid **(A)**. FTIR spectrum of benzendicarboxylic acid, calcium, and calcium impregnated with benzenedicarboxylic acid **(B)**, and X-ray diffraction pattern of benzendicarboxylic acid, calcium, and calcium impregnated with benzenedicarboxylic acid. Benzendicarboxylic acid (Bd), Calcium (Cal), and calcium impregnated with benzenedicarboxylic acid (Bd_Cal_) **(C)**.

Transmission electron micrograph revealed a single crystalline Ca (44 μm) impregnated with Bd. The comparison of the three FTIR spectra (i.e., Bd, Ca, and Bd_Ca_) had one unique dejection at 2,237 in Bd_Ca_, which was absent in Bd and Ca, showing a stable bond between them. The XRD assay came up with the two characteristic peaks in Bd, one in Ca, and three in Bd_Ca_, showing the sustained identity of the two impregnated substances (Figure 1).

### Determination of Growth Attributes

The results of this study showed that Bd alone had the potential to boost the growth parameters of the plants, i.e., root fresh weight, shoot fresh weight, root dry weight, and shoot dry weight under multiple stress conditions of DDT toxicity and Cd stress. All these growth credentials were improved 1.3–1.9 times after Bd treatment. However, another interesting finding was the positive support of C toward the plant growth parameters. Growth parameters were elevated by 1.3–1.6 times due to Ca use. The growth regulator Bd allowed *Brassica* plants to overcome DDT toxicity, introducing pollutant tolerance in them. Besides, it also elevated the plant growth parameters up to 162% (Supplementary Figure 1). However, Ca could enhance the plant growth, up to 131%, when exposed to DDT. The maximum growth enhancement (258%) was reported in the plants exposed to Bd_Ca_, and it could be reduced up to 221% under DDT toxicity. In contrast, the toxic effects of DDT caused a 34–49% reduction in growth attributes and leaf area of the plants as opposed to control therapy. Bd, Ca, and Bd_Ca_ were proved successful in augmenting chlorophyll contents in *Brassica* plants. However, enhancement in chlorophyll contents was recorded as 139% by Bd application, 125% by Ca procedure, and 158% by Bd_Ca_. DDT stress plants also displayed a substantial increase of 147% in chlorophyll contents when primed with Bd_Ca_. Cd toxicity exerts deleterious effects on *Brassica* plants, retarding 18% of growth attributes, 21% of leaf area, and 15% chlorophyll contents (Supplementary Figure 2).

### Estimation of Leaf Relative Water Content

**FIGURE 2:**
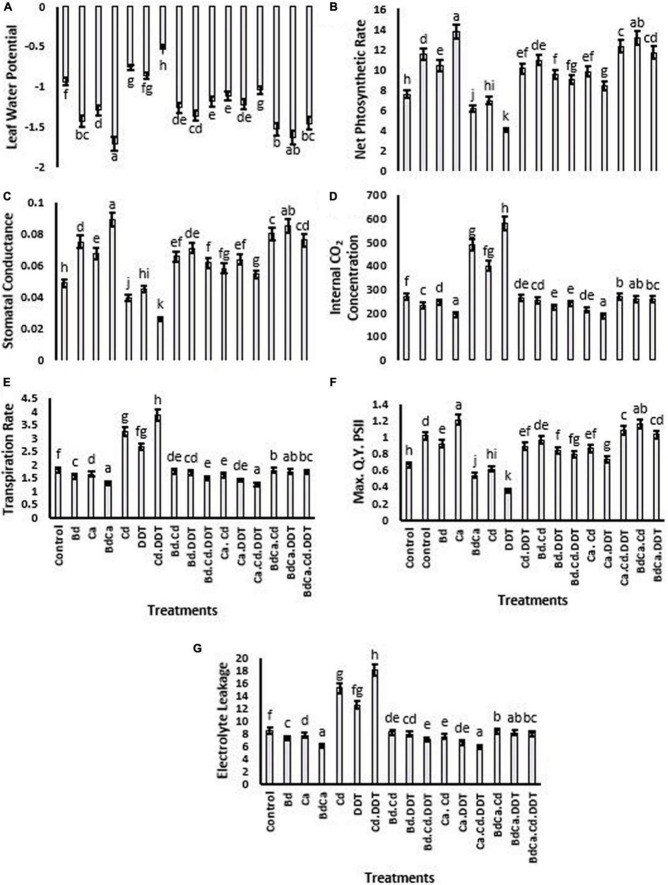
Effect of benzenedicarboxylic acid (Bd) on photosynthesis-related parameters of *Brassica alboglabra* under toxic conditions of dichlorodiphenyltrichloroethane (DDT) and cadmium (Cd). Leaf relative water content **(A)**, net photosynthesis rate **(B)**, stomatal conductance **(C)**, internal CO_2_ concentration **(D)**, transpiration rate **(E)**, maximum quality yield of PS-II **(F)**, and electrolyte leakage **(G)**. Values demonstrate means ± SD (*n* = 5). Different letters indicate a significant difference among the treatments (*P* ≤ 0.05). Bd, benzenedicarboxylic acid; Cd, cadmium; DDT, dichlorodiphenyltrichloroethane; Bd_Cal_, benzedicarboxylic acid doped calcium.

The findings showed the impact of Cd and DDT on LRWC (53.24 and 18.61%, respectively) relative to the *Brassica* plant. The maximum LRWC was recorded in the negative Cd control treatments. However, as opposed to the control, the Bd treatment increased the value of LRWC (46.31%). Ca application to *Brassica* plants also increased LRWC by 36.98%, while under Bd_Ca_ treatment, a 51.76% increase in LRWC was evident. The treatment with DDT reduced *Pn* to 51.37%. A significant increase in *Pn* due to Bd and Ca was found at 25.61% and 22.13%, respectively. The Bd_Ca_ application reported a maximum *Pn* increase of 40.25%. As far as the photosystem-quenching Fv/Fm is concerned, the effect of Bd-impregnated Ca treatment was significantly higher than the individual effects of Ca and Bd (28.66%). Bd_Ca_ was the most successful plant treatment that produced the greatest improvement in stomatal conductance, *Ci*, and the rate of transpiration. However, all these physiological parameters were negatively affected by the stress caused by Cd and DDT toxicity. The cell physiology of *Brassica* was positively affected by both Bd and Ca (Figure 2).

### Determination of Photosynthetic Pigments and Soluble Sugars

The DDT toxicity substantially decreased photosynthetic pigments by up to 46.31% as compared to the control treatment. Bd induced an elevation of 1.35 times in the pigments and Ca treatment of 1.24 times. Bd_Ca_ lied in the stronger support to the overall chlorophyll contents, raising it to 235.78%. A 17.92% reduction in chlorophyll in the Kale plants was caused by Cd. DDT was responsible for a substantial decrease in the soluble sugar content of 3.18 mg/g when compared to the control treatment of 5.48 mg/g. Bd (12.06 mg/g) and Ca treatment (9.92 mg/g) recorded the substantial mitigation of the soluble sugar contents. While, in the presence of DDT, Bd_Ca_ increased the soluble sugar content up to (12.38 mg/g), whereas, in the absence of toxic DDT, the contents were increased up to 14.69 mg/g. In terms of soluble sugars, Cd stress had deleterious effects on *Brassica* plants. However, Bd_Ca_ (mg/g) could successfully mitigate the detrimental effects of the plant stressors (Table 1).

**TABLE 1:** Effect of benzenedicarboxylic acid (Bd) and calcium nanoparticles (Ca) on chlorophyll *a*, chlorophyll *b*, total chlorophyll, and soluble sugars of *Brassica alboglabra* under Cd stress, temperature stress, and BDE-28 toxicity.

Treatments	Chl*a*	Chl*b*	Total Chlorophyll	Soluble Sugars (mg g^–1^)
Control	0.95 ± 0.05*i*	0.52 ± 0.08*h*	1.48 ± 0.16*i*	5.48 ± 0.19*h*
Bd	1.45 ± 0.04*c*	0.80 ± 0.04*b*	2.26 ± 0.48*bc*	12.06 ± 0.24*bc*
Ca	1.31 ± 0.05*e*	0.72 ± 0.03*d*	2.04 ± 0.029*e*	9.92 ± 0.11*d*
Bd_Ca_	1.73 ± 0.03*a*	0.95 ± 0.01*a*	2.69 ± 0.36*a*	14.69 ± 0.19*a*
Cd	0.77 ± 0.04*kl*	0.42 ± 0.064*jk*	1.20 ± 0.54*kl*	2.79 ± 0.32*jk*
DDT	0.87 ± 0.03*k*	0.48 ± 0.04*j*	1.36 ± 0.049*k*	3.18 ± 0.41*j*
Cd.DDT	1.50 ± 0.02*l*	1.28 ± 0.04*k*	1.78 ± 0.075*l*	3.41 ± 0.17*l*
Bd.Cd	1.27 ± 0.04*e*	0.70 ± 0.03*de*	1.98 ± 0.067*e*	9.37 ± 0.18*de*
Bd.DDT	1.38 ± 0.06*ef*	0.75 ± 0.01*de*	2.15 ± 0.052*ef*	9.75 ± 0.21*de*
Bd.Cd.DDT	1.20 ± 0.03*h*	0.66 ± 0.07*fg*	1.86 ± 0.047*h*	7.07 ± 0.17*fg*
Ca. Cd	1.13 ± 0.04*g*	0.62 ± 0.06*fg*	1.76 ± 0.036*g*	7.23 ± 0.17*fg*
Ca.DDT	1.24 ± 0.03*gh*	0.68 ± 0.04*f*	1.92 ± 0.075*gh*	7.62 ± 0.16*f*
Ca.Cd.DDT	1.05 ± 0.05*ij*	0.58 ± 0.06*hi*	1.64 ± 0.15*ij*	4.93 ± 0.18*hi*
Bd_Ca_.Cd	1.55 ± 0.08*b*	0.85 ± 0.07*bc*	2.41 ± 0.094*b*	12.00 ± 0.14*bc*
Bd_Ca_ DDT	1.65 ± 0.04*bc*	0.90 ± 0.03*b*	2.58 ± 0.042*bc*	12.38 ± 0.24*b*
Bd_Ca_.Cd.DDT	1.47 ± 0.07*d*	0.81 ± 0.04*de*	2.29 ± 0.075*d*	9.70 ± 0.19*de*

*Values demonstrate means ± SD (n = 5). Different letters indicate significant difference among the treatments (P ≤ 0.05). C, control; Cd, Cadmium; Ca, Calcium.*

### Antioxidant Defenses

**FIGURE 3:**
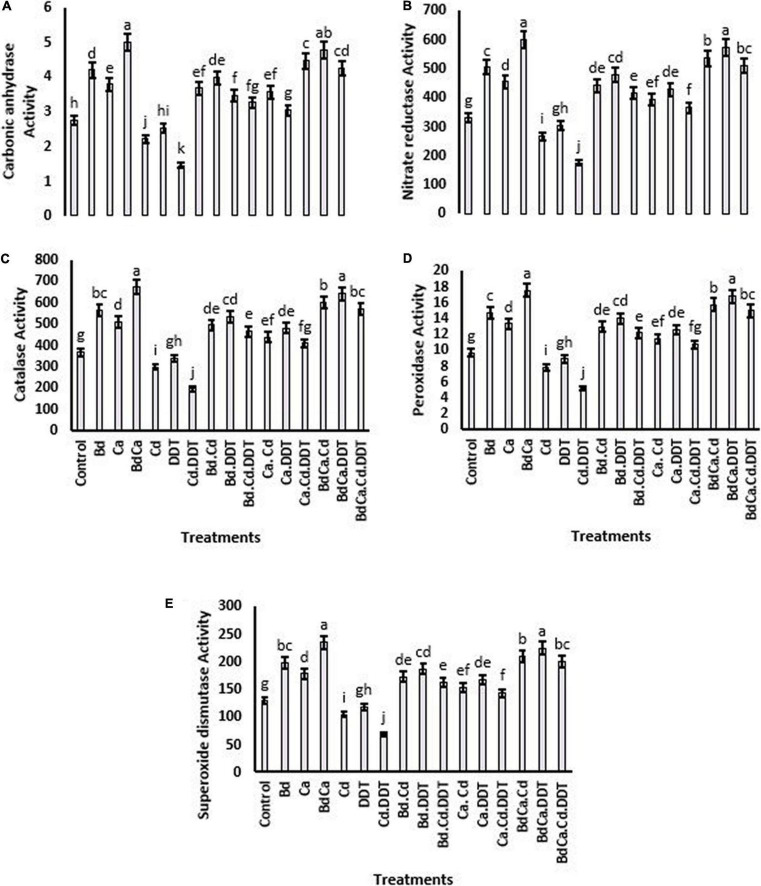
Effect of benzenedicarboxylic acid (Bd) on antioxidant defense parameters of *Brassica alboglabra* under under toxic environment of dichlorodiphenyltrichloroethane (DDT) and cadmium (Cd). Carbonic anhydrase **(A)**, nitrate reductase activity **(B)**, catalase activity **(C)**, peroxidase activity **(D)**, and superoxide dismutase activity **(E)**. Values demonstrate means ± SD (*n* = 5). Different letters indicate a significant difference among the treatments (*P* ≤ 0.05). Bd, benzenedicarboxylic acid; Cd, cadmium; DDT, dichlorodiphenyltrichloroethane; Bd_Cal_, benzedicarboxylic acid doped calcium.

Nitrate reductase activity was significantly decreased by the toxic content of DDT and increased by the application of Bd and Ca. Bd_Ca_ resulted in the most successful mitigation, which increased the activity of the enzyme by 247.64%. The enzyme activity was adversely affected by Cd, and Bd_Ca_ significantly mitigated Cd and DDT toxicity, resulting in an increase in nitrate reductase activity of 218.39%. Similar patterns have been observed for other enzymes studied, such as catalase, POD, SOD, and carbon anhydrase. DDT toxicity and Cd variations adversely affected the activities of the enzymes. However, Bd_Ca_ effectively alleviated the hazardous effects and increased the activity of the enzymes by up to 227.36%, improving the antioxidant protection of the plant (Figure 3).

### Electrolyte Leakage Malondialdehyde Content (MDA) and H_2_O_2_

The DDT toxicity and Cd stress boosted the EL. Due to DDT, an increase of 234.74% was reported in EL. However, in comparison with the control treatment, the increased EL due to Cd was significantly elevated. Under the toxic effects of DDT, malondialdehyde content (MDA) was also increased by more than two times. The H_2_O_2_ content was elevated by toxic DDT and Cd (223.54% and 208.36%, respectively) by strictly following the identical pattern. Successfully downregulated H_2_O_2_ contents by Bd and Ca treatments minimized the risk of oxidative harm to plant cells (Figure 3).

### Enzymatic Analysis

The enzymatic content of monodehydroascorbate reductase (MDHAR), dehydroascorbate reductase (DHAR), glutathione-*S*-transferase (GST), glutathione peroxidase (GPX), glutathione reductase (GR), and APX in Kale plants was significantly increased. The enzyme activities were also improved by Ca treatment alone, but the level of upregulation was significantly lower than that of Bd. The amounts of all these enzymes shared a common pattern of elevation after exposure to Ca and Bd. Whereas significant reductions in the enzyme activities were observed after their exposure to the plant stressors, i.e., Cd and DDT. The mitigating function of Bd_Ca_, however, was dominant, and it mitigated all the stress effects of Cd and DDT on *B. alboglabra* (Table 2).

**TABLE 2:** Effect of benzenedicarboxylic acid (Bd) and calcium nanoparticles (Ca) on dehydroascorbate reductase (DHAR), monodehydroascorbate reductase (MDHAR), glutathione-s-transferase (GST), glutathione reductase (GR), glutathione peroxidase (GPX), and ascorbate peroxidase (APX), in *Brassica alboglabra* under Cd stress, temperature stress, and BDE-28 toxicity.

Treatments	DHAR (nmol min^–1^ mg^–1^ protein)	MDHAR (nmol min^–1^ mg^–1^ protein)	GST (nmol g^–1^ FW)	GR (EU mg^–1^ protein)	GPX (μmol g^–1^ FW)	APX (EU mg^–1^ protein)
Control	103.00 ± 3.9*h*	54.20 ± 2.47*h*	37.10 ± 1.25*h*	3.98 ± 0.12*h*	53.14 ± 1.34*f*	2.21 ± 0.08*f*
Bd	157.59 ± 7.1*c*	82.93 ± 3.44*bc*	56.76 ± 1.27*bc*	6.09 ± 1.01*d*	81.30 ± 2.91*c*	3.38 ± 0.15*bc*
Ca	142.14 ± 5.61*d*	74.80 ± 2.1*d*	51.20 ± 1.87*d*	5.49 ± 0.13*e*	73.33 ± 1.75*d*	3.05 ± 0.05*d*
Bd_Ca_	187.46 ± 2.8*a*	98.64 ± 1.11*a*	67.52 ± 1.9*a*	7.24 ± 0.03*a*	96.71 ± 0.99*a*	4.02 ± 0.02*a*
Cd	83.43 ± 3.67*jk*	43.90 ± 2.21*jk*	30.05 ± 0.81*jk*	3.22 ± 0.34*j*	43.04 ± 2.23*g*	1.79 ± 0.07*g*
DDT	94.76 ± 7.14*j*	49.86 ± 2.21*j*	34.13 ± 1.17*j*	3.66 ± 0.41*hi*	48.89 ± 1.43*fg*	2.03 ± 0.05*fg*
Cd.DDT	55.59 ± 3.1*k*	29.73 ± 1.14*k*	19.66 ± 0.99*k*	2.11 ± 0.05*k*	28.16 ± 0.64*h*	1.17 ± 0.08*h*
Bd.Cd	138.02 ± 8.62*de*	72.63 ± 2.32*de*	49.71 ± 1.14*de*	5.33 ± 0.28*ef*	71.21 ± 1.28*de*	2.96 ± 0.11*de*
Bd.DDT	149.35 ± 6.61*de*	78.59 ± 2.41*de*	53.80 ± 1.53*de*	5.77 ± 0.32*de*	77.05 ± 2.15*cd*	3.20 ± 0.08*cd*
Bd.Cd.DDT	129.78 ± 5.34*fg*	68.29 ± 2.14*fg*	46.75 ± 2.19*fg*	5.01 ± 0.31*f*	66.96 ± 3.17*e*	2.78 ± 0.06*e*
Ca. Cd	122.57 ± 4.74*fg*	64.50 ± 2.71*fg*	44.15 ± 1.22*fg*	4.74 ± 0.37*fg*	63.24 ± 1.98*e*	2.63 ± 0.09*e*
Ca.DDT	133.90 ± 3.67*f*	70.46 ± 1.34*f*	48.23 ± 1.77*f*	5.17 ± 0.61*ef*	69.08 ± 3.75*de*	2.87 ± 0.05*de*
Ca.Cd.DDT	114.33 ± 3.67*hi*	60.16 ± 3.11*hi*	41.18 ± 2.07*hi*	4.42 ± 1.01*g*	58.99 ± 1.63*a*	2.45 ± 0.09*g*
Bd_Ca_.Cd	167.89 ± 3.67*b*	88.35 ± 2.49*bc*	60.47 ± 1.33*b*	6.49 ± 0.11*c*	86.62 ± 0.64*b*	3.60 ± 0.07*b*
Bd_Ca_ DDT	179.22 ± 3.67*c*	94.31 ± 2.42*b*	64.55 ± 2.16*bc*	6.93 ± 0.03*ab*	92.46 ± 1.38*ab*	3.85 ± 0.07*ab*
Bd_Ca_.Cd.DDT	159.65 ± 3.67*de*	84.01 ± 2.21*de*	57.51 ± 2.84*de*	6.17 ± 0.34*cd*	82.40 ± 2.41*bc*	3.43 ± 0.06*bc*

*Values demonstrate means ± SD (n = 5). Different letters indicate significant difference among the treatments (P ≤ 0.05). C, control; Cd, Cadmium; Ca, Calcium.*

### Proline, Lycopene, β-Carotene, and Lutein Contents

**FIGURE 4:**
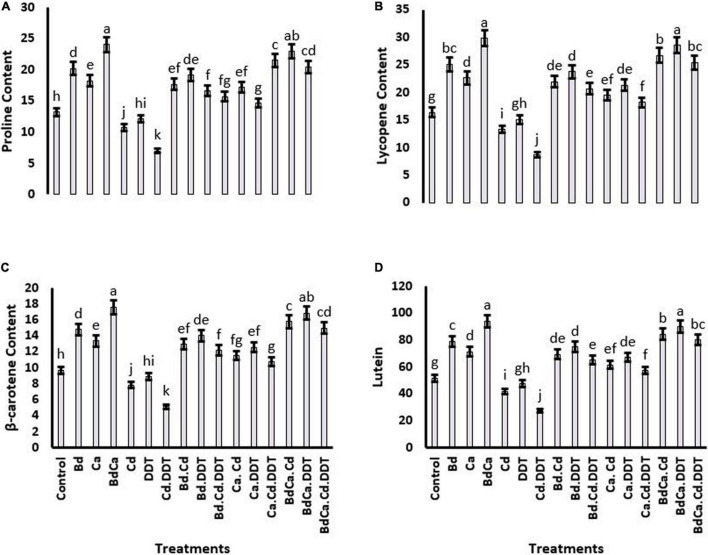
Effect of benzenedicarboxylic acid (Bd) on physiological parameters of *Brassica alboglabra* under under toxic environment of dichlorodiphenyltrichloroethane (DDT) and cadmium (Cd). Proline content **(A)**, lycopene content **(B)**, β-carotene content **(C)**, and lutein content **(D)**. Values demonstrate means ± SD (*n* = 5). Different letters indicate a significant difference among the treatments (*P* ≤ 0.05). Bd, benzenedicarboxylic acid; Cd, cadmium; DDT, dichlorodiphenyltrichloroethane; Bd_Cal_, benzedicarboxylic acid doped calcium.

Proline contents showed significant retardation in the plants treated with DDT (43.71%). However, a positive effect of Bd was recorded on the proline, resulting in a significant elevation of 46.18% in its contents as compared to the control treatment. The positive results of Bd on proline contents were also comparable with the positive results of Ca. Cd toxicity negatively affected lycopene, β-carotene, and lutein. Cd and DDT jointly abridged the proline contents more severely. However, all these biochemicals were substantially improved by the three treatments, namely, Bd, Ca, and Bd_Ca_ (Figure 4).

### Water Potential, Leaf Osmotic Potential, and MSI

Exogenously applied Bd caused the leaf water potential of Kale plants to increase by 153.620%. Kale plants are grown in a toxic DDT environment, which, however, had an 8.69% lower potential for leaf water. Similarly, Cd application negatively impacted the leaf water potential and caused a significant reduction, whereas Ca and Bd_Ca_ treatments have demonstrated significant improvements in leaf water potential. The treatments also mitigated the Cd and DDT toxicity effectively. The leaf osmotic potential and MSI were also found following a similar pattern. The stress effects of Cd and DDT toxicity were successfully mitigated by Bd_Ca_, which was the best treatment used in the assays (Table 3).

**TABLE 3:** Effect of benzenedicarboxylic acid (Bd) and calcium nanoparticles (Ca) on water potential, leaf osmotic potential, membrane stability index (MSI), root and shoot Cd uptake, translocation factor (TF) of Cd and metal tolerance index (MTI) of Cd in *Brassica alboglabra* under Cd stress, temperature stress, and BDE-28 toxicity.

Treatments	Water potential	Leaf osmotic potential	MSI	Root Cd (μg g^–1^)	Shoot Cd (μg g^–1^)	Leaf Cd (μg g^–1^)	Seed Cd (μg g^–1^)	TF_Cd_	MTI	RE_Cd_ (%)
Control	0.69 ± 0.04*h*	0.860 ± 0.04*h*	0.93 ± 0.03*f*	–	–	–	–	–	–	–
Bd	1.06 ± 0.19*d*	1.316 ± 0.03*d*	1.42 ± 0.04*c*	–	–	–	–	–	–	–
Ca	0.95 ± 0.03*e*	1.187 ± 0.02*e*	1.28 ± 0.01*d*	–	–	–	–	–	–	–
Bd_Ca_	1.26 ± 0.1*a*	1.565 ± 0.04*a*	1.69 ± 0.01*a*	–	–	–	–	–	–	–
Cd	0.56 ± 0.03*j*	0.697 ± 0.01*j*	0.75 ± 0.01*g*	22.21 ± 0.15*b*	20.41 ± 0.03*b*	19.89 ± 0.15*b*	19.35 ± 0.03*b*	633.60 ± 5.01*b*	7.87 ± 3.5*b*	13.09 ± 0.08*b*
DDT	0.63 ± 0.04*hi*	0.791 ± 0.07*hi*	0.86 ± 0.04*fg*	–	–	–	–	–	–	–
Cd.DDT	0.37 ± 0.03*k*	0.456 ± 0.03*k*	0.49 ± 0.02*h*	26.41 ± 0.21*a*	24.27 ± 0.2*a*	23.65 ± 0.21*a*	23.01 ± 0.2*a*	753.28 ± 8.04*a*	5.15 ± 3.1*a*	8.085 ± 0.51*a*
Bd.Cd	0.92 ± 0.03*ef*	1.152 ± 0.04*ef*	1.25 ± 0.03*de*	11.97 ± 0.1*cd*	11.00 ± 0.1*cd*	10.72 ± 0.1*cd*	10.43 ± 0.1*cd*	341.44 ± 0.01*cd*	13.01 ± 6.9*cd*	67.76 ± 1.24*cd*
Bd.DDT	1.00 ± 0.03*de*	1.247 ± 0.03*de*	1.35 ± 0.02*cd*	–	–	–	–	–	–	–
Bd.Cd.DDT	0.87 ± 0.04*f*	1.084 ± 0.03*f*	1.17 ± 0.01*e*	10.24 ± 0.48*de*	9.41 ± 0.01*d*	9.17 ± 0.48*de*	8.92 ± 0.01*de*	292.16 ± 0.03*d*	12.23 ± 5.8*de*	49.28 ± 1.33*d*
Ca. Cd	0.82 ± 0.02*fg*	1.023 ± 0.01*fg*	1.11 ± 0.02*e*	10.98 ± 0.38*d*	10.09 ± 0.02*cd*	9.83 ± 0.38*d*	9.57 ± 0.02*d*	313.28 ± 0.01*cd*	11.55 ± 3.7*d*	55.055 ± 2.02*cd*
Ca.DDT	0.90 ± 0.04*ef*	1.118 ± 0.02*ef*	1.21 ± 0.01*de*	–	–	–	–	–	–	–
Ca.Cd.DDT	0.77 ± 0.04*g*	0.955 ± 0.02*g*	1.03 ± 0.01*a*	8.51 ± 0.19*f*	7.82 ± 0.01*e*	7.62 ± 0.19*f*	7.42 ± 0.21*f*	242.88 ± 0.01*e*	10.78 ± 6.1*f*	36.575 ± 1.14*e*
Bd_Ca_.Cd	1.12 ± 0.03*c*	1.402 ± 0.02*c*	1.52 ± 0.01*b*	12.22 ± 0.38*c*	11.23 ± 0.21*c*	10.94 ± 0.38*c*	10.64 ± 0.03*c*	348.48 ± 0.03*c*	15.83 ± 2.96*c*	90.09 ± 2.35*c*
Bd_Ca_ DDT	1.20 ± 0.03*ab*	1.496 ± 0.01*ab*	1.62 ± 0.02*ab*	–	–	–	–	–	–	–
Bd_Ca_.Cd.DDT	1.07 ± 0.03*cd*	1.333 ± 0.03*cd*	1.44 ± 0.02*bc*	11.72 ± 0.4*cd*	10.77 ± 0.03*cd*	10.50 ± 0.4*cd*	10.21 ± 0.01*cd*	334.40 ± 0.03*cd*	15.05 ± 6.3*cd*	71.61 ± 3.14*cd*

*Values demonstrate means ± SD (n = 5). Different letters indicate significant difference among the treatments (P ≤ 0.05). C, control; Cd, Cadmium; Ca, Calcium.*

### Cd Contents

The Cd contents were recorded the highest in root tissues of the plants (22.21 μg/g), which were reduced to 20.41 μg/g in the shoots, 19.89 μg/g in the leaves, and ultimately 19.35 μg/g in the seeds when Kale plants were exposed to Cd. The TF value of Cd was 633.60, and MTI of Kale against Cd was 7.87, with a removal efficacy (RE) value of 13.09. DDT put the stress effects on Kale plants, significantly increasing the Cd contents in the plant tissues, elevated TF, decreased MTI, and reduced RE. The best performing treatment Bd_Ca_ showed reduced Cd content of 11.72 μg/g in the plant roots, 10.77 μg/g in shoots, 10.50 μg/g in leaves, and 10.21 μg/g in seeds under the influence of two stressors, namely, Cd and DDT. The treatment also showed a TF value of 334.40, an MTI value of 15.05, and an RE value of 71.61 (Table 3).

### DDT Contents, TF, and Pollutant Tolerance Index

**FIGURE 5:**
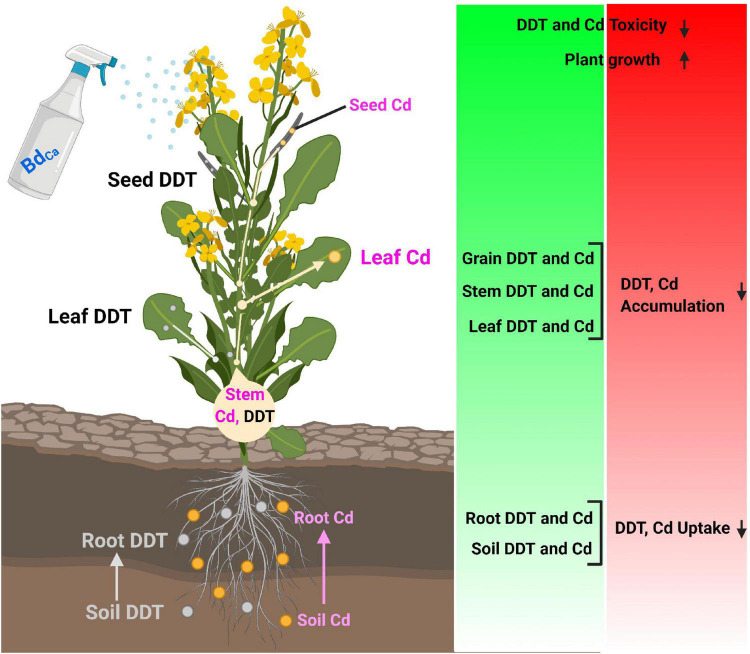
Illustration of mechanistic role of calcium nanoparticles impregnated with benzenedicarboxylic acid in alleviation of combined stress of DDT and Cd in Brassica alboglabra by modulating bioaccumulation, antioxidative machinery and osmoregulators.

The DDT accumulation was higher in roots (8.39 μg/g) compared to shoots (5.59 μg/g), leaves (3.71 μg/g), and seeds (1.69 μg/g) under the DDT control treatment during the study. Meanwhile, the TF value was 182.04, along with the MTI value of 16.87 and the RE value of 173.28. Cd played the role of an enhancer for DDT accumulations (elevating DDT bioconcentration) in both plant tissues and seeds (2.44 μg/g). Both the Ca and Bd mitigated the stress caused by DDT and Cd and lowered down the DDT contents in plant tissues. The Cd application assisted the stress effects of DDT and caused a significant increase in its bioaccumulation in plant tissues. After the application of Bd, the DDT contents were recorded 5.33 μg/g in roots, 3.55 μg/g in shoots, 2.36 μg/g in leaves, and 1.07 μg/g in seeds of the Kale plants. It could reduce the TF value up to 115.62, whereas it improved the MTI value up to 26.59 and the RE value up to 584.82. The most promising treatment was Bd_Ca_ that did not allow DDT contents to exceed 5.39 μg/g in the roots, 3.59 μg/g in the shoots, 2.38 μg/g in the leaves, and 1.08 μg/g in the seeds of the Kale plants. It recorded a TF value of 116.85 with improved MTI and RE values (Table 4). The overall observation of the experiments was summarized in a schematic diagram to understand the positive effects of Bd_Ca_ and the mechanistic aspects behind them (Figure 5).

**TABLE 4:** Effect of benzenedicarboxylic acid (Bd) and calcium nanoparticles (Ca) on water potential, leaf osmotic potential, membrane stability index (MSI), root and shoot Cd uptake, translocation factor (TF) of Cd and pollutant tolerance index (PTI) of Cd in *Brassica alboglabra* under Cd stress, temperature stress, and BDE-28 toxicity.

Treatments	Root DDT (μg g^–1^)	Shoot DDT (μg g^–1^)	Leaf DDT (μg g^–1^)	Seed DDT (μg g^–1^)	TF_DDT_	PTI	RE_DDT_ (%)
Control	–	–	–	–	–	–	–
Bd	–	–	–	–	–	–	–
Ca	–	–	–	–	–	–	–
Bd_Ca_	–	–	–	–	–	–	–
Cd	–	–	–	–	–	–	–
DDT	8.39 ± 0.19*b*	5.59 ± 0.1*b*	3.71 ± 0.15*b*	1.69 ± 0.02*b*	182.04 ± 4.47*b*	16.87 ± 6.9*g*	173.28 ± 3.56*g*
Cd.DDT	12.13 ± 0.21*a*	8.09 ± 0.2*a*	5.37 ± 0.21*a*	2.44 ± 0.2*a*	263.22 ± 7.63*a*	9.72 ± 3.1*h*	75.81 ± 4.37*h*
Bd.Cd	–	–	–	–	–	–	–
Bd.DDT	5.33 ± 0.43*cd*	3.55 ± 0.01*cd*	2.36 ± 0.4*cd*	1.07 ± 0.01*cd*	115.62 ± 3.17*cd*	26.59 ± 6.3*c*	584.82 ± 4.74*c*
Bd.Cd.DDT	4.71 ± 0.48*d*	3.14 ± 0.01*d*	2.08 ± 0.48*cd*	0.95 ± 0.01*cd*	102.09 ± 4.03*d*	23.11 ± 5.8*de*	462.08 ± 5.31*de*
Ca. Cd	–	–	–	–	–	–	–
Ca.DDT	4.42 ± 0.38*de*	2.95 ± 0.03*de*	1.96 ± 0.38*d*	0.89 ± 0.03*d*	95.94 ± 2.13*de*	23.84 ± 6.1*d*	465.69 ± 9.11*d*
Ca.Cd.DDT	3.91 ± 0.1*e*	2.61 ± 0.21*e*	1.73 ± 0.38*de*	0.79 ± 0.21*de*	84.87 ± 1.17*e*	20.36 ± 3.5*f*	342.95 ± 8.06*f*
Bd_Ca_.Cd	–	–	–	–	–	–	–
Bd_Ca_ DDT	5.44 ± 0.15*c*	3.63 ± 0.03*c*	2.41 ± 0.1*c*	1.09 ± 0.03*c*	118.08 ± 1.98*c*	31.91 ± 2.96*a*	794.2 ± 5.73*a*
Bd_Ca_.Cd.DDT	5.39 ± 0.38*cd*	3.59 ± 0.02*cd*	2.38 ± 0.19*cd*	1.08 ± 0.1*cd*	116.85 ± 2.44*cd*	28.43 ± 3.7*b*	671.46 ± 4.24*b*

*Values demonstrate means ± SD (n = 5). Different letters indicate significant difference among the treatments (P ≤ 0.05). C, control; Cd, Cadmium; Ca, Calcium.*

## Discussion

For the first time, the current investigation sets out a method for the sustainable exogenous delivery of Bd to a plant surface after Ca impregnation. Bd is a fungal metabolite primarily known for its role in the augmentation of cell defenses against pathogenic fungi (Ahmad et al., 2014a,b, 2019). The reaction of the plant to Bd is purely dose- and duration-dependent. The early studies reported that this compound improved the nutritional profiles of tomato fruits. However, with the high water solubility, it cannot be applied to plant surfaces in rainy and humid environments. To ensure the sustained availability of Bd at plant surfaces, a formulation development is necessary. This study remains an outstanding approach in the field of plant protection and cell physiology. In addition, Bd has a tendency to change the physiological processes and genetic controls of plants (Khan et al., 2019; Ahmad et al., 2020a).

Stress factors (e.g., DDT) induce metabolic imbalances and encourage EL and oxidative stress, contributing to the accumulation of solutes in the form of adaptive cellular responses (Jan et al., 2012; Shafique et al., 2014). This lays the basis for the increased coefficient of bioconcentration of DDT in various *Brassica* plant tissues. In addition, the analysis first defines the TF of DDT in Kale plants, elaborating the fate of the pollutant absorbed into the edible plant (Rignell-Hydbom et al., 2007; Khan et al., 2017). Bd is involved in initializing the expression of the genetic cascade associated with starch and sugar, which essentially determines the energy economy of the plant. This property of Bd has been used during stress conditions to harmonize the physiological balances. In addition, the scientific research investigated the mitigation actions of Ca and Bd through the reharmonization of stress markers, such as EL, LRWC, gaseous exchange, antioxidant defenses, and the activities of enzymes. The primary tools of environmental toxicants to slow plant growth and metabolism are EL, *Ci*, reduced *Pn*, and leaf water potential. Cd toxicity also exerts similar stress symptoms on the physiology of plants (Faiz et al., 2021). The study recommends Bd_Ca_, which has the potential to rehabilitate cellular homeostasis, as an effective plant metabolism modulator. It was evident that Bd_Ca_ increased the photosynthetic pigments (Chl *a* and Chl *b*), *Pn*, and total soluble sugars, which demonstrated the full-swing rehabilitation of Kale photosynthetic function, which was reduced by Cd and DDT. During the current investigation, increased chlorophyll biosynthesis subsequently increased the photosynthetic activity and augmented development in Bd_Ca_-applied plants.

The least objective of a plant is to ensure its survival through the devastating period of stress, and the least objective of a farmer is to get enough yield to cover the cultivation costs (Yasin et al., 2018b). Both of these objectives can be achieved by improving plant tolerance against stressors (Zaheer et al., 2016). Therefore, this study considered the tolerance index against both the pollutant DDT and the metal Cd. Both the stress mitigators (i.e., Ca, Bd) and their combined form (Bd_Ca_) successfully achieved phoenix tolerance indices DDT and Cd. Removing the pollutants and heavy metals is the safest method for remediating environmental toxins and safeguarding the food chain for consumers, including humans (Shah et al., 2020). It also makes the toxins and pollutants to be exposed to environmental deteriorations for natural degradation (Liu et al., 2013; Abbas et al., 2020). This study introduced a unique technique based on Bd-impregnated Ca, which successfully hampered the entry of DDT into the food chain, which would certainly be detoxified overtime under natural degradation.

In addition, the single and combined effect of Ca and Bd resulted in beneficial physiochemical changes in plant cells. A few studies have previously used Ca for topical delivery of RNAi to make plants immune to pathogens (Mitter et al., 2017). Even, there was no ultimate conclusion about how the application of nanoparticles will respond to plant growth. This study highlighted the supporting role of Ca nanoparticles in plant growth for the first time. All the tested growth parameters of the Kale plants were in favor of the technique. It also elevated the enzyme activities responsible for antioxidant defenses, metabolic processes, and other physiological responses without a negative effect on plant growth. The study showed that Ca had an enormous potential to be used under stress conditions as a plant growth enhancer. A beneficial aspect of Ca explored in this study is getting no adverse cross talk with Bd.

The importance of LRWC serves as a biomarker of plant-water relationships (Wang et al., 2019; Ahmad et al., 2021a). There are detrimental effects of Cd stress on both osmotic ability and water (Khan et al., 2018; Hafeez et al., 2020). The LRWC values of *Brassica* plants were also negatively impacted by Cd, suggesting decreased drought tolerance. All the three treatments, namely, Ca, Bd, and Bd_Ca_, greatly improved LRWC during the current investigation, proving a good option for high precipitation areas and areas adversely affected by Cd contamination. Lipid peroxidation-induced membranous injuries deteriorate cell metabolic processes and serve as a biomarker of cellular injuries in stressed plants. Similarly, the increased amount of ROS triggers the necrosis of cells in stressed plants (Arshad et al., 2016; Tariq et al., 2021). However, under stress, Ca and Bd-applied plants showed a decreased synthesis of ROS (H_2_O_2_). Furthermore, the amount of MDA, which is the final by-product of lipid peroxidation, was decreased by Bd_Ca_ supplementation. Similarly, oxidative damage to cells and lipid membranes is triggered by a higher H_2_O_2_ level in stressed plants (Farooq et al., 2013; Sattar et al., 2020). The EL caused by the weakened cell membrane decreases the development of plant growth and biomass. Several studies have shown that the upregulated antioxidant machinery activity decreases the synthesis of H_2_O_2_ in stressed plants (Farooq et al., 2013; Sattar et al., 2020; Ahmad et al., 2021b). Superoxide is detoxified by the action of SOD into a less injurious H_2_O_2_, which is eventually transformed by the activity of POD, catalse (CAT), and APX into H_2_O. By improving the function of the antioxidative system in stressed plants, the exogenous application of Bd_Ca_ improved stress tolerance. In addition to the decreased level of EL, H_2_O_2_, and MDA, the upregulation in antioxidant machinery has highlighted the favorable role of Bd_Ca_ in reducing oxidative injuries and controlling redox homeostasis in stressed plants.

The SOD is known as one of the key antioxidant defenses of plant cells, which is essential for survival under the conditions of stress (Askary et al., 2017). While SOD and CAT are primarily localized in the plant cell peroxisomes and mitochondria, they have shown a significant response to stress conditions (Shereefa and Kumaraswamy, 2016). The stress responses in Kale plants, however, were mitigated by Bd_Ca_ treatment. A comprehensive interrelation between the enzyme activities of Bd, Ca, Bd_Ca_, ROS, MDA, and antioxidants is given in the current investigation. It can be inferred that all three treatments (i.e., Bd, Ca, and Bd_Ca_) have improved plant defenses against Cd stress and DDT toxicity by considering the overall physiological responses of the plant. However, in this respect, the most promising formulation was Bd_Ca_. The scavenging of H_2_O_2_ was a primary feature of Kale plants thriving under stress conditions in the plant cells. The key source of ROS is H_2_O_2_, and one of the most important substrates for scavenging H_2_O_2_ is ascorbate (Ibrahim et al., 2016; Nahar et al., 2017). The greater biosynthesis of ROS than that which is scavenged (by the antioxidant system) contributes to irreversible oxidative damages to the plant cells. The non-enzymatic antioxidant protection mechanism contains ascorbate, glutathione, and carotenoids. However, the main components of the enzymatic antioxidant mechanism are GST, GR, GPX, DHAR, MDHAR, APX, SOD, and CAT. A dual role to improve the oxidative defense mechanism of Kale plants has been performed by Bd_Ca_. It raised the activity of antioxidants to further increase the protective layers of plants against oxidative damages.

Researchers have long been involved in increasing the proline and carotenoid content of edible plants (i.e., β-carotene, lycopene, and lutein). The pigmented elements of plants that contribute to the photosynthetic machinery and perform a defensive role against photodamage are carotenoids (Tan and Norhaizan, 2019). Foods rich in carotenoids defend humans against age-related illnesses. Lycopene is a major carotenoid of Kale plants with a distinctive red color and unique antioxidant properties. Proline is a primary component of the machinery for protein biosynthesis that determines the cell structure, metabolism, profile of nutrition, wound healing, antioxidant reactions, and responses to protection (Wu et al., 2011; Shaddad et al., 2013). Proline has a pivotal role in the stability of the arrangement of macromolecules and the turgidity of the structure of the cell membrane in stressed plants. Proline scavenges ROS in plants and attenuates tension. Bd_Ca_ successfully increased the proline and carotenoid contents in Kale plants under various stress conditions during this study. It hardened the plants and made them tolerate DDT toxicity and Cd stress. The increased concentration of DDT in plant roots growing in a toxic DDT environment is because vacuole and root cell wall can accumulate and retain higher POP contents compared to the shoot cells. Due to modified homeostasis, the exogenous application of Bd_Ca_ decreased the DDT content in plants. Furthermore, all the advantages of reducing the content of DDT in Kale leaves and Kale seeds are the achievements of the study. Reducing the toxins and contaminants in the seeds of the Kale plants will be of much assistance to prevent humans from DDT. Given the essence of transfer, persistence, bioaccumulation, and lethal effects of DDT, the treatment of Bd_Ca_ achieved a hallmark of preventing the entry into the human food chain. In addition, the application of Bd_Ca_ decreased the bioaccumulation of the Kale roots, hampering the transfer of DDT from soil to food at the first level. The analysis can be established to look for the bioconcentration activity of POPs in other food plants.

This study discloses a novel strategy to grow healthier food crops in a contaminated environment. The large-scale production and application of Bd_Ca_ can mitigate chemical stress and heavy metal stress, and *Brassica* produces more crop yields with the least accumulation of POPs. It can be a very beneficial technique of crop cultivation in the contaminated soil and air. However, some detailed studies are required to verify the effects of Bd_Ca_ on some other crop plants.

## Conclusion

It can be concluded that the growth and physiological attributes of Kale were successfully improved by Ca- and Bd-dependent Bd_Ca_ under various stress conditions caused by Cd and DDT pollution. By reducing the biosynthesis of ROS, enhancing the antioxidative mechanism, and modulating the synthesis of osmoregulators, the exogenously applied Bd_Ca_ increased plant resistance to the combined abiotic stressors. In addition, Bd_Ca_ inhibited the absorption of DDT in plant tissues and decreased its TF. Therefore, the novel treatment of Bd_Ca_ is recommended for relieving multiple stresses and for a stainable crop production system. However, further field experiments are compulsory to evaluate the efficacy of Bd_Ca_ in Kale plants and other crop plants.

## Data Availability Statement

The original contributions presented in the study are included in the article/Supplementary Material, further inquiries can be directed to the corresponding author/s.

## Author Contributions

SM and IS: perform experiments. AA and WA: design research. IS: perform WS statistical analysis. NY: writing. AS: review and drafting. MS and SA: writing and funding acquisition. All authors contributed to the article and approved the submitted version.

## Conflict of Interest

The authors declare that the research was conducted in the absence of any commercial or financial relationships that could be construed as a potential conflict of interest.

## Publisher’s Note

All claims expressed in this article are solely those of the authors and do not necessarily represent those of their affiliated organizations, or those of the publisher, the editors and the reviewers. Any product that may be evaluated in this article, or claim that may be made by its manufacturer, is not guaranteed or endorsed by the publisher.
