# Supplementary material for: Calcium Nanoparticles Impregnated With Benzenedicarboxylic Acid: A New Approach to Alleviate Combined Stress of DDT and Cadmium in Brassica alboglabra by Modulating Bioacummulation, Antioxidative Machinery and Osmoregulators
Source: Front Plant Sci. 2022 Mar 9;13:825829. doi: 10.3389/fpls.2022.825829 (PMC8959818; doi:10.3389/fpls.2022.825829)
Supplement: Supplementary file 1 [file Data_Sheet_1.docx]

# **Calcium nanoparticles impregnated with benzenedicarboxylic acid a new approach to alleviate combined stress of DDT and cadmium in *Brassica alboglabra* by modulating bioacummulation, antioxidative machinery and osmoregulators**

Samavia Mubeen ^1^, Iqra Shahzadi ^2^, Waheed Akram ^3^, Wajid Saeed ^4^, Nasim Ahmad Yasin ^5^, Aqeel Ahmad ^6^, Anis Ali Shah ^7^, Manzer H. Siddiqui ^8^

1. State Key Laboratory for Biocontrol, School of Life Sciences, Sun Yat-Sen University, Guangzhou-510275, China.

2. Hubei Key Laboratory of Biomass Resource Chemistry and Environmental Biotechnology, Hubei International Scientific and Technological Cooperation Base of Sustainable Resource and Energy, Hubei Engineering Center of Natural Polymers-based Medical Materials, School of Resource and Environmental Science, Wuhan University, Wuhan, 430079, China

3. Department of Plant Pathology, Institute of Agricultural Sciences, University of the Punjab, Lahore, Punjab, Pakistan.

4. Key laboratory of crop cultivation and farming System, Agriculture College, Guangxi University,Nanning China

5. Senior Superintendent Garden, University of the Punjab, Lahore

6. Key Laboratory of Land Surface Pattern and Simulation, Institute of Geographic Sciences and Natural Resources Research, Chinese Academy of Sciences, Beijing, 100101, China

7. Department of Botany, University of Narowal, Narowal, Pakistan.

8. Department of Botany and Microbiology, College of Science, King Saud University, Riyadh, 11451, Saudi Arabia.

**Corresponding Author:**

[anisalibot@gmail.com](mailto:anisalibot@gmail.com); [aqeelahmad1@gmail.com](mailto:aqeelahmad1@gmail.com)

**Methods**

**Determination of Growth Attributes**

The harvested plants were separated into the root, shoot, and leaf samples. These plant parts were kept individually in distilled water for 60 s. Afterward, plant parts were dried on blotting paper and stored at -80°C. Fresh weight of roots and shoots together with their relevant lengths were recorded. Then, different parts of the plants were dried in an oven at 80°C for 48 h to determine growth attributes produced in various parts.

**Estimation of Leaf Relative Water Content**

Leaf relative water content (*LRWC*) was estimated from leaves of 5 plants (3 leaves plant^-1^). The separated leaves were packed in a plastic bag and brought instantly to the lab for estimation of *LRWC* according to the method of Ahmad et al. ^1^.

### Determination of Photosynthetic pigments

The amount of total carotenoid and chlorophyll contents (Chl *a* and Chl *b*) were evaluated by grounding 100 mg fresh weight (FW) of foliage sample along with 8 mL of acetone 80% (v/v) in a pre-chilled mortar. The mixture of the extract was filtered, and the volume adjusted to 10 mL by adding cold acetone. The spectrophotometric value of the solution was observed at 470, 663.2, and 646.8 nm ^2^.

**Assessment of Soluble Sugars**

The phenol sulphuric acid method was employed for the evaluation of soluble sugars, as described by Dubois et al. ^3^. A homogenized 0.5 g plant sample was added in test tubes containing 80% ethanol (10 mL). This solution was heated over a water bath at 80 °C for 60 min, and the mixture (0.5 mL) was transferred to other tubes. An equal volume of deionized water along with 1 mL of 18% phenol was mixed. Tubes were allowed to cool at room temperature for 30 min after which absorbance was recorded at 490 nm. The contents of total sugars present in the sample were estimated according to the following equation:

$$Sugar=\left( \frac{Sample absorbance\times dilution factor\times K value}{weight of fresh plant tissue} \right)$$

**Assessment of Gas Exchange Parameters and net Photosynthetic Rate**

The transpiration (*E*) rate, stomatal conductance (*Gs*), and net photosynthesis (*Pn*) rate were observed (between 09:30 and 12:00 am) from the second fully opened leaf with the help of portable gas exchange system (Li-COR Inc., Biosciences, Lincoln: NE, USA).

**Estimation of Cd Contents**

The powder obtained from the oven-dried plant sample was sieved through a 100 nylon mesh. Then 0.6 g powder was digested by using 10 mL HNO_3_ by employing a microwave digestion apparatus and diluted to 50 mL with deionized water, followed by an assessment of total Cd via flame atomic absorption spectrophotometer.

**Metal Tolerance Index**

Metal tolerance index (MTI) from plant biomass was calculated by employing the following formula of Deng et al. ^4^:

MTI=DW _treated plant_/DW _control_.

The translocation factor (TF) of Cd from root to or shoot was determined according to the following equation ^5^:

TF= Cd concentration in shoot/ Cd concentration in root

**Evaluation of Hydrogen Peroxide**

For estimation of H_2_O_2_ content, about 0.2 g plant sample was cut into pieces and homogenized with 5 mL of 0.1% (w/v) TCA in an ice-chilled mortar. After centrifugation of homogenate at 12,000 x g for 15 min at 4°C, the resulting supernatant (0.5 mL) was mixed with 1 mL of KI (1 M) and 0.5 mL of potassium phosphate buffer (10 mM) at pH 7.0. The spectrophotometric value of the solution was observed at 390 nm, and H_2_O_2_ quantity was estimated by comparing with the standard curve of known concentrations of H_2_O_2_ ^6^.

**Assessment of Lipid Peroxidation and Electrolyte Leakage**

The level of lipid peroxidation was estimated from thiobarbituric acid (TCA) reaction through computing the quantity of malondialdehyde (MDA), according to Hodges et al. ^9^. The MDA content was estimated at 532 nm and deducted from the spectrophotometric value at 600 nm. The level of electrolyte leakage (EL) from fully expanded leaf was measured as described by Tian et al. ^10^.

**Measurement of Leaf Osmotic Potential**

The osmotic potential from the cell sap of ﬂag leaves was measured by using a freezing point osmometer, according to Capell and Doerﬄing ^11^. To obtain cell sap, the foliage sample (50 μl) was enclosed in a plastic syringe placed at 0°C. Afterward, it was pressed to discharge cell sap from the alleviated leaf. Readings were taken from osmometer in mosmol kg^-1^ and converted to osmotic potential (MPa) with the help of Van’t Hoffs’ equation as follows:

ψS(MPa) = mOsmol x GC (0.831) x 10^-5^ x Temperature (^o^K)

GC= Gas constant= 0.831x 10^-5^(R)

T (K) = Thermodynamic temperature

**Membrane stability index (MSI)**

The electrical conductivity of different treatments was measured for the estimation of membrane stability index ^12^. Prewashed leaf sample (100 mg) was placed in 2 sets of test tubes enclosing 10 mL of distilled water. One set was heated in a water bath at 40°C for 0.5 h, and the other set was heated in a water bath at 100 °C for 10 min. The electrical conductivities (C1 and C2) of these sets were noted, correspondingly. MSI was measured from the following equation:

MSI (%) = {1-(C1/C2)} × 100; where C1 and C2 were electrical conductivity at 40 and 100 °C, respectively.

**Estimation of Water Potential**

Scholander pressure chamber was used for the evaluation of leaf water potential ^13^. Flag leaf was placed over the holder of the pressure vessel. The pressure was generated inside the pressure vessel until the cell sap appeared at the cut side of the leaf. The value of the generated pressure is equivalent to the negative force by which water is held in plant tissues. It was analyzed and deliberated in MPa.

**Evaluation of Antioxidant Enzymatic Activities**

The antioxidant enzymes including monodehydroascorbate reductase (MDHAR, EC 1.6.5.4), POX (EC 1.11.1.7), dehydroascorbate reductase (DHAR), ascorbate peroxidase (APX), catalase (CAT), glutathione reductase (GR), glutathione-S-transferase (GST), superoxide dismutase (SOD) and glutathione peroxidases (GPX) were determined.

With the help of MagNA Lyser and 1 mM ASC (Roche, Vilvoorde, Belgium), fresh leaves sample (100 mg) was homogenized along with solutions including 0.25% (v/v) Triton X-100, 10% (w/v) polyvinylpyrrolidone (PVP), 1 mM phenylmethylsulfonyl fluoride (PMSF),1 mL of 50 mM potassium phosphate buffer at pH 7.0. The decrease of NBT (nitroblue tetrazolium) was measured at 560 nm to estimate SOD activity ^14^. The oxidation of pyrogallol (ε_430_ = 2.47 mM^-1^ cm^-1^) was analyzed for the determination of POX activity, according to Schurt et al. ^15^. The amount of H_2_O_2_ decomposed at 240 nm was estimated to assess CAT activity ^16^. Peroxidase (POD) activity was measured using the absorbance coefficient 26,600 l M^-1^ cm^-1^ at 470 nm wavelength ^16^. Murshed et al. ^17^ was used for estimation of GR, APX, DHAR, and MDHAR activities. The conjugation of GSH through additional 1-chloro-2, 4-dinitrobenzene (CDNB) was accomplished, and absorbance was observed at 340 nm (ε_340_ = 0.0096 μM^-1^ cm^-1^ for evaluation of GST activity according to Yang et al., ^18^. The reduction in NADPH was observed to evaluate GPX activity at 340 nm (ε_340_ = 6.22 mM^-1^cm^-1^), according to Horvath et al. ^19^.

**Assessment of Proline Content**

The quantity of proline contents was estimated, according to Bates et al. ^20^. For this purpose, a 0.2 g dry leaf sample was vortexed with 10 mL of 3% (w/v) aqueous sulfosalicylic acid prior to centrifugation of homogenate at 10,000g for 10 min. The resulting supernatant was passed through Whatman No 2 filter paper and 1 mL filtrate was homogenized with an equivalent amount of glacial acetic acid and ninhydrin reagent (1.25 g ninhydrin, 30 mL of glacial acetic acid, 20 mL 6 M H_3_PO_4_), and placed over ice bath at 100 °C. After 60 min, tubes were placed in an ice bath, and 3 mL toluene was mixed. After 50 min, the spectrophotometric value was observed at 520 nm and compared with the standard curve.

**Lycopene Estimation**

For lycopene estimation, the HPLC method recommended by Choksi and Joshi ^21^ was strictly followed.

**Carotenoids (B-Carotene and Lutein)**

For Carotenoids estimation, the method of Yu et al. ^22^ was followed. Approximately 200 mg of plant tissue was pulverized in 3 ml extraction solvent (hexane, acetone, and ethanol; 2:1:1) by rapidly shaking for 30 min in a scintillation vial containing a steel rod. The sample was centrifuged for 10 min at 1,800 g, and the supernatant collected. The pellet was washed with another 3 ml extraction solvent, and the supernatant collected and pooled. The solvent was removed by evaporation at room temperature under a stream of nitrogen gas. Triacyl glycerides were saponified in the residue by heating at 80°C for 1 h in 5 ml methanolic-KOH (10% w/v KOH in methanol: water [80:20 v/v]). Carotenoids and aqueous compounds were partitioned using 2 ml H2O and 3 ml petroleum ether. The ether phase and two 3 ml ether washings were collected, pooled, and the solvent evaporated at room temperature under a nitrogen gas stream. Their residue was resuspended in 200µl of acetonitrile: methylene chloride: methanol (50:40:10 [v/v]) with 0.5% (w/v) butylated hydroxytoluene and filtered through a 0.2µm pore size nylon syringe filter into an HPLC sample vial. The extract was immediately analyzed using HPLC. Aliquots of 20µl were loaded onto a 4.6µm·250 mm reverse-phase C30YMC ‘‘Carotenoid Column’’ (Waters Ltd, Mississauga, ON, Canada) at 35°C. Mobile phases consisted of methanol (A) and tert-methyl butyl ether (B). A linear gradient starting at 95% A and5% B, proceeding to 35% A and 65% B over25 min and a flow rate of 1.2 ml min–1was used for elution. Compounds in the eluate were monitored at450 nm using a photodiode array. Peaks were identified by their retention time and absorption spectra compared to those of known standards(CaroteNature, Switzerland). Quantification of carotenoids (B-Carotene and Lutein) was conducted using curves constructed with authentic standards.

**Measurement of Carbonic Anhydrase Activity**

Carbonic Anhydrase Activity was measured according to our previous study Ahmad et al. ^23^

**Assessment of Nitrate Reductase Activity**

Method of Khan et al. ^24^ was followed to assess nitrate reductase activity.

**References:**

1. Ahmad P, Ahanger MA, Alyemeni MN, et al. Zinc application mitigates the adverse effects of NaCl stress on mustard [ Brassica juncea (L.) Czern & Coss] through modulating compatible organic solutes, antioxidant enzymes, and flavonoid content. *J Plant Interact*. 2017;12(1):429-437. doi:10.1080/17429145.2017.1385867

2. Lichtenthaler HK. [34] Chlorophylls and carotenoids: Pigments of photosynthetic biomembranes. In: ; 1987:350-382. doi:10.1016/0076-6879(87)48036-1

3. DuBois M, Gilles KA, Hamilton JK, Rebers PA, Smith F. Colorimetric Method for Determination of Sugars and Related Substances. *Anal Chem*. 1956;28(3):350-356. doi:10.1021/ac60111a017

4. Deng DM, Shu WS, Zhang J, et al. Zinc and cadmium accumulation and tolerance in populations of Sedum alfredii. *Environ Pollut*. 2007;147(2):381-386. doi:10.1016/j.envpol.2006.05.024

5. Dos Santos Utmazian MN, Wieshammer G, Vega R, Wenzel WW. Hydroponic screening for metal resistance and accumulation of cadmium and zinc in twenty clones of willows and poplars. *Environ Pollut*. 2007;148(1):155-165. doi:10.1016/j.envpol.2006.10.045

6. Loreto F, Velikova V. Isoprene Produced by Leaves Protects the Photosynthetic Apparatus against Ozone Damage, Quenches Ozone Products, and Reduces Lipid Peroxidation of Cellular Membranes. *Plant Physiol*. 2001;127(4):1781-1787. doi:10.1104/pp.010497

7. Shereefa LAH, Kumaraswamy M. Reactive oxygen species and ascorbate–glutathione interplay in signaling and stress responses in Sesamum orientale L. against Alternaria sesami (Kawamura) Mohanty and Behera. *J Saudi Soc Agric Sci*. 2016;15(1):48-56. doi:10.1016/j.jssas.2014.04.007

8. Hasanuzzaman M, Nahar K, Hossain MS, et al. Coordinated actions of glyoxalase and antioxidant defense systems in conferring abiotic stress tolerance in plants. *Int J Mol Sci*. 2017;18(1):200. doi:10.3390/ijms18010200

9. Hodges DM, DeLong JM, Forney CF, Prange RK. Improving the thiobarbituric acid-reactive-substances assay for estimating lipid peroxidation in plant tissues containing anthocyanin and other interfering compounds. *Planta*. 1999;207(4):604-611. doi:10.1007/s004250050524

10. Tian S, Guo R, Zou X, et al. Priming With the Green Leaf Volatile (Z)-3-Hexeny-1-yl Acetate Enhances Salinity Stress Tolerance in Peanut (Arachis hypogaea L.) Seedlings. *Front Plant Sci*. 2019;10. doi:10.3389/fpls.2019.00785

11. Capell B, Dörffling K. Genotype-specific differences in chilling tolerance of maize in relation to chilling-induced changes in water status and abscisic acid accumulation. *Physiol Plant*. 1993;88(4):638-646. doi:10.1111/j.1399-3054.1993.tb01383.x

12. Chakraborty U, Pradhan B. Oxidative stress in five wheat varieties (Triticum aestivum L.) exposed to water stress and study of their antioxidant enzyme defense system, water stress responsive metabolites and H2O2 accumulation. *Brazilian J Plant Physiol*. 2012;24(2):117-130. doi:10.1590/S1677-04202012000200005

13. Scholander PF, Bradstreet ED, Hemmingsen EA, Hammel HT. Sap Pressure in Vascular Plants: Negative hydrostatic pressure can be measured in plants. *Science (80- )*. 1965;148(3668):339-346. doi:10.1126/science.148.3668.339

14. Liu N, Lin Z, Guan L, Gaughan G, Lin G. Antioxidant Enzymes Regulate Reactive Oxygen Species during Pod Elongation in Pisum sativum and Brassica chinensis. Rahman A, ed. *PLoS One*. 2014;9(2):e87588. doi:10.1371/journal.pone.0087588

15. Schurt DA, Cruz MFA, Nascimento KJT, Filippi MCC, Rodrigues FA. Silicon potentiates the activities of defense enzymes in the leaf sheaths of rice plants infected by Rhizoctonia solani. *Trop Plant Pathol*. 2014;39(6):457-463. doi:10.1590/S1982-56762014000600007

16. Konieczny R, Banaś AK, Surówka E, Michalec Ż, Miszalski Z, Libik-Konieczny M. Pattern of antioxidant enzyme activities and hydrogen peroxide content during developmental stages of rhizogenesis from hypocotyl explants of Mesembryanthemum crystallinum L. *Plant Cell Rep*. 2014;33(1):165-177. doi:10.1007/s00299-013-1520-4

17. Murshed R, Lopez-Lauri F, Sallanon H. Effect of water stress on antioxidant systems and oxidative parameters in fruits of tomato (Solanum lycopersicon L, cv. Micro-tom). *Physiol Mol Biol Plants*. 2013;19(3):363-378. doi:10.1007/s12298-013-0173-7

18. Yang X, Wei J, Wu Z, Gao J. Effects of Substrate-Binding Site Residues on the Biochemical Properties of a Tau Class Glutathione S-Transferase from Oryza sativa. *Genes (Basel)*. 2019;11(1):25. doi:10.3390/genes11010025

19. Horváth E, Bela K, Gallé Á, et al. Compensation of Mutation in Arabidopsis glutathione transferase (AtGSTU) Genes under Control or Salt Stress Conditions. *Int J Mol Sci*. 2020;21(7):2349. doi:10.3390/ijms21072349

20. Bates LS, Waldren RP, Teare ID. Rapid determination of free proline for water-stress studies. *Plant Soil*. 1973;39(1):205-207. doi:10.1007/BF00018060

21. Choksi PM, Joshi VY. A Review on Lycopene—Extraction, Purification, Stability and Applications. *Int J Food Prop*. 2007;10(2):289-298. doi:10.1080/10942910601052699

22. Yu B, Lydiate DJ, Young LW, Schäfer UA, Hannoufa A. Enhancing the carotenoid content of Brassica napus seeds by downregulating lycopene epsilon cyclase. *Transgenic Res*. 2008;17(4):573-585. doi:10.1007/s11248-007-9131-x

23. Ahmad A, Khan TA, Mubeen S, et al. Metabolic and Proteomic Perspectives of Augmentation of Nutritional Contents and Plant Defense in Vigna unguiculata. *Biomolecules*. 2020;10(2):224. doi:10.3390/biom10020224

24. Khan MIR, Khan NA, Masood A, Per TS, Asgher M. Hydrogen Peroxide Alleviates Nickel-Inhibited Photosynthetic Responses through Increase in Use-Efficiency of Nitrogen and Sulfur, and Glutathione Production in Mustard. *Front Plant Sci*. 2016;7. doi:10.3389/fpls.2016.00044

**Table S1:** List of treatment combinations of Cd stress, temperature stress, and DDT toxicity to *Brassica alboglabra*.

| Treatments | Description |
| --- | --- |
| Control | Standard growth solution |
| Bd | Benzenedicarboxylic acid |
| Cd | Cadmium |
| DDT | Dichlorodiphenyltrichloroethane |
| Bd.Cd | Benzenedicarboxylic acid+ Cadmium |
| Bd.DDT | Benzenedicarboxylic acid + Dichlorodiphenyltrichloroethane |
| Cd.DDT | Cadmium+ Dichlorodiphenyltrichloroethane |
| Bd.Cd.DDT | Benzenedicarboxylic acid + Cadmium+ Dichlorodiphenyltrichloroethane |
| Bd.Cd.DDT.T15 | Benzenedicarboxylic acid + Cadmium+ Dichlorodiphenyltrichloroethane +15°C (2h) |
| Bd.Cd.DDT.T35 | Benzenedicarboxylic acid + Cadmium+ Dichlorodiphenyltrichloroethane + 35°C (2h) |
| Bd.Cd.DDT.T45 | Benzenedicarboxylic acid + Cadmium+ Dichlorodiphenyltrichloroethane + 45°C (2h) |
| Bd.Cd.DDT.T50 | Benzenedicarboxylic acid + Cadmium+ Dichlorodiphenyltrichloroethane + 50°C (2h) |


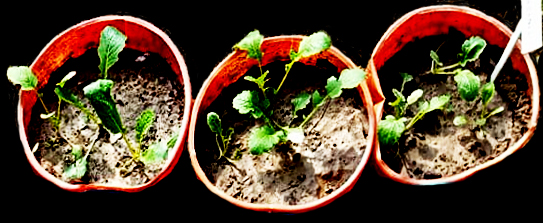

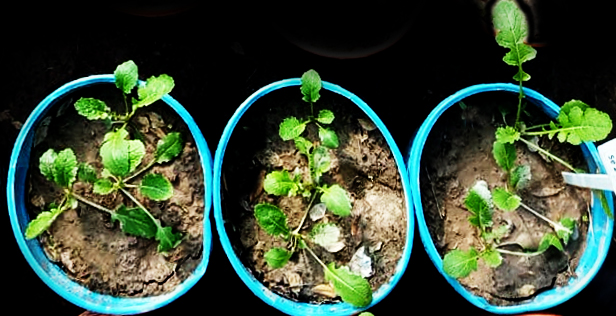

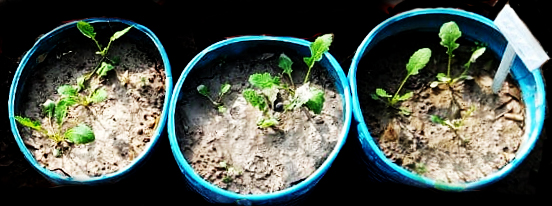

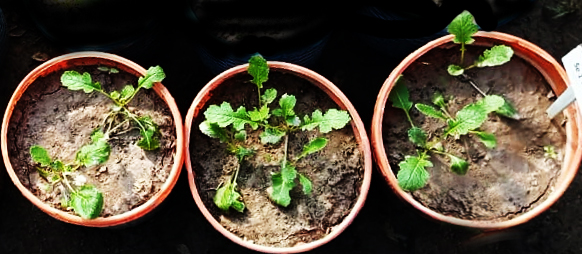


**(D)**

**(A)**

**(C)**

**(B)**

**Figure S1:** Pictorial representation of the treatment impact on plants. The treatments were applied in a combination of positive control having Bd_Ca_ . DDT. Cd (A), Bd_Ca_ (B), DDT.Cd (C), and negative control plants (D)

**(H)**

**(G)**

**(F)**

**(E)**

**(D)**

**(B)**

**(A)**

**(C)**

**Figure S2:** Effect of benzenedicarboxylic acid and calcium nanoparticles (Ca) on growth attributes of *Brassica alboglabra* under Cd stress, and DDT toxicity. Root length (A), Shoot length (B), Root fresh mass (C), Shoot fresh mass (D), Root dry mass (E), Shoot dry mass (F), Leaf area (G), SPAD chlorophyll value *Y*:0.78*X*+19.7 (H). Values demonstrate means ± SD (n=5). Different letters indicate significant difference among the treatments (*P*≤0.05). Bd= Benzenedicarboxylic acid; Cd= Cadmium, DDT= Dichlorodiphenyltrichloroethane, Bd_Ca_= benzedicarboxylic acid doped calcium.
